# Clinical outcomes of laparoscopic versus open right hepatectomy for liver tumors: A meta-analysis

**DOI:** 10.1097/MD.0000000000018667

**Published:** 2020-01-03

**Authors:** Qiang Hong, Jianjun Wang, Yong Wang, Baojuan Fu, Yuejun Fang, Qin Tong, Tao Liu, Zhangqiang Wu, Junchao Zhou

**Affiliations:** a Department of Surgical Oncology, Jinhua GuangFu Oncology Hospital; b Department of General Surgery, Sir Run Run Shaw Hospital, College of Medicine, Zhejiang University, Hangzhou; c Key Laboratory of Laparoscopic Technology of Zhejiang Province, Sir Run Run Shaw Hospital, College of Medicine, Zhejiang University, Hangzhou, Zhejiang, China.

**Keywords:** laparoscopic, liver resection, right hepatectomy

## Abstract

**Background:**

Laparoscopic right hepatectomy (LRH) is one of the most challenging procedures. Right liver resections have been always performed in open procedure and open right hepatectomy (ORH) was initially considered as routine way. Moreover, it is unclear how beneficial the minimally invasive technique is to patients; thus, we conducted a meta-analysis to acquire a more reliable conclusion about the feasibility and safety of LRH compared with ORH.

**Methods:**

We comprehensively searched the electronic databases of PubMed, Embase, and the Cochrane Library using the key words. Meta-analysis was performed using the Review Manager, with results expressed as odds ratio and weighted mean difference with 95% confidence intervals. The fixed-effect model was selected initially if high heterogeneity was not present between the studies; otherwise, the randomized-effect model was used. Subgroup analysis was performed based on different surgical methods of pure laparoscopic operation or hand-assisted operation.

**Results:**

Seven studies with 467 patients were included. In the overall analysis, less intraoperative blood loss (MD = –155.17; 95% CI, –238.89, –71.45; *P* = .0003) and a shorter length of stay (MD = –4.45; 95% CI, –5.84, –3.07; *P* < .00001) were observed in the LRH group compared to the ORH group. There were fewer overall complications (OR = 0.30; 95% CI, 0.10, 0.90; *P* = 0.03) and severe complications (OR = 0.24; 95% CI, 0.10, 0.58; *P* = .002;) in the LRH group than in the ORH group. The disadvantage of LRH was the longer operative time (MD = 49.39; 95% CI, 5.33, 93.45; *P* = .03). No significant difference was observed between the 2 groups in portal occlusion, rate of R0 resection, transfusion rate, mild complications, and postoperative mortality. In the subgroup analysis, intraoperative blood loss was significantly lower in the pure LRH group and hand-assist LRH group compared with ORH group. Length of stay was shorter by use of pure LRH and hand-assisted LRH manners than ORH. The incidence rate of complications was lower in the pure LRH group than in the ORH group. In contrast, there was no significant difference between hand-assisted LRH group and ORH group.

**Conclusion:**

Compared to ORH, LRH has short-term surgical advantages and leads to a shorter recovery time in selected patients. We speculate that the operative time of LRH is closer with ORH. Overall, LRH can be considered a feasible choice in routine clinical practice with experienced surgeons, although more evidence is needed to make a definitive conclusion.

## 1 Introduction

For the past decades, minimally invasive operation has increasingly improved. As a representative technique of minimally invasive operation, the laparoscopic technique is skillfully performed in the area of hepatobiliary surgery.^[1–5]^ According to previous studies, the preliminary results of laparoscopic minor liver resection have been achieved.^[6–9]^ As for left lateral sectionectomy, laparoscopic method is preferred because resulting injuries are minor and complications rare.^[10]^ However, major liver resection is one of the most challenging procedures, and the high tendency of morbidity and postoperative liver failure limit its clinical application in the field of laparoscopic operation. Moreover, pervasive underlying liver diseases such as virus hepatitis with hepatic cirrhosis sharply increase the surgical difficulty.^[11]^

Right liver resections have always been performed in the conventional manner, and they were initially considered unsuitable for laparoscopy; however, an increasing number of laparoscopic right liver resections have been reported,^[12–14]^ and the evaluation of feasibility and clinical outcomes from different studies remains inconsistent. Therefore, we conducted a meta-analysis to compare laparoscopic and open right hepatectomy (ORH) that assess the feasibility, morbidity, and mortality of these 2 different surgical methods.

## 2 Methods

### 2.1 Search strategy and selection criteria

We searched for published articles comparing laparoscopic and ORH from Pubmed, Embase, and the Cochrane library up to February 2019 using the following medical subject heading terms: ‘laparoscope’, ‘hepatectomy’, and ‘laparotomy’. Additionally, the following keywords were added to the search plan: ‘celioscopy’, ‘peritoneoscopy’, and ‘major liver resection’. References from the included studies were searched for additional studies using the same afore mentioned methods. No language restrictions were applied to the search.

The following types of studies were included:

(1) original articles;
(2) comparative studies of laparoscopic and ORH, including retrospective studies, cohort studies, and randomized controlled trials (RCTs);
(3) studies of patients with benign and malignant tumors; and
(4) studies for which the original data could be extracted.

Ethical approval was not necessary because this study did not involve patient consent.

### 2.2 Data extraction and quality assessment

Two reviewers read the full articles independently and identified whether the studies could be included according to the aforementioned criteria. This work was then reevaluated and checked by a senior researcher. The all measured results of the included studies was divided into 2 categories:

(1) intraoperative outcomes (blood loss, hepatic portal occlusion and blood transfusion);
(2) postoperative outcomes (length of stay, overall complication, R0 resection, morbidity and mortality).

The postoperative morbidity was categorized according to the Clavien-Dindo classification. Grade I and Grade II complications are categorized as minor complication, and major complication refers to Grade III to V complications.

### 2.3 Assessment of quality and bias risk

The data quality of non-randomized studies was assessed using Newcastle Ottawa quality assessment scale (NOS) by examining the following 2 factors: patient selection, comparability of the study groups, and assessment of outcome.^[15]^ Maximum scores in the selection, comparability, and outcome categories were 4, 2, and 3, respectively. The summation of scores of the 2 categories was evaluated to assess the quality of retrieved studies. A study with a total score of 7–9, 5–6, and 0–4 was defined as good, fair and poor, respectively.

### 2.4 Statistical analysis

We used odds ratio (OR) to compare dichotomous variables, and all results are reported with 95% confidence intervals (CIs). Continuous variables were assessed by weighted mean differences (WMDs) with a 95% CI. When the statistical data were reported as a median and range, the method by Hozo et al^[16]^ was used to transform the data into a mean and standard deviation. Both binary and continuous data were calculated using the random and fixed-effect model. The fixed-effect model was selected initially if high heterogeneity was not present between the studies; otherwise, the random-effect model was used.^[17]^ Heterogeneity between studies was evaluated using the chi-square test and *I*^2^ test, with significance set at *P* < .05. *I*^2^ values between 0% and 25%, above 25%, and above 75% suggest low, moderate and high heterogeneity, respectively. If the standard deviation was not available, it was calculated according to the guidelines of the Cochrane Collaboration. Forest plots were used for graphic presentation of the results. We used the Begg and Egger tests to assess publication bias among the studies. Asymmetry of the funnel plot and *P* < .05 from the Eggers test indicate evidence of publication bias. Subgroup analysis was performed based on different surgical methods of pure laparoscopic operation or hand-assisted operation with the clinical outcome of laparoscopic right hepatectomy (LRH) and ORH. Sensitivity analysis was conducted to assess robustness of outcomes by serial omission of each study. Statistical analysis was performed using Review Manager, version 5.2 software (Cochrane Collaboration), whereas Begg and Egger test was performed with STATA 13 SE (StataCorp LP). To avoid false-positive outcomes, false discovery rate (FDR) correction method was applied to adjust *P* value using R software version 3.4.3. FDR-corrected *P*_adjusted_ < .05 from the association test was considered statistically significant.

## 3 Results

**Figure 1:**
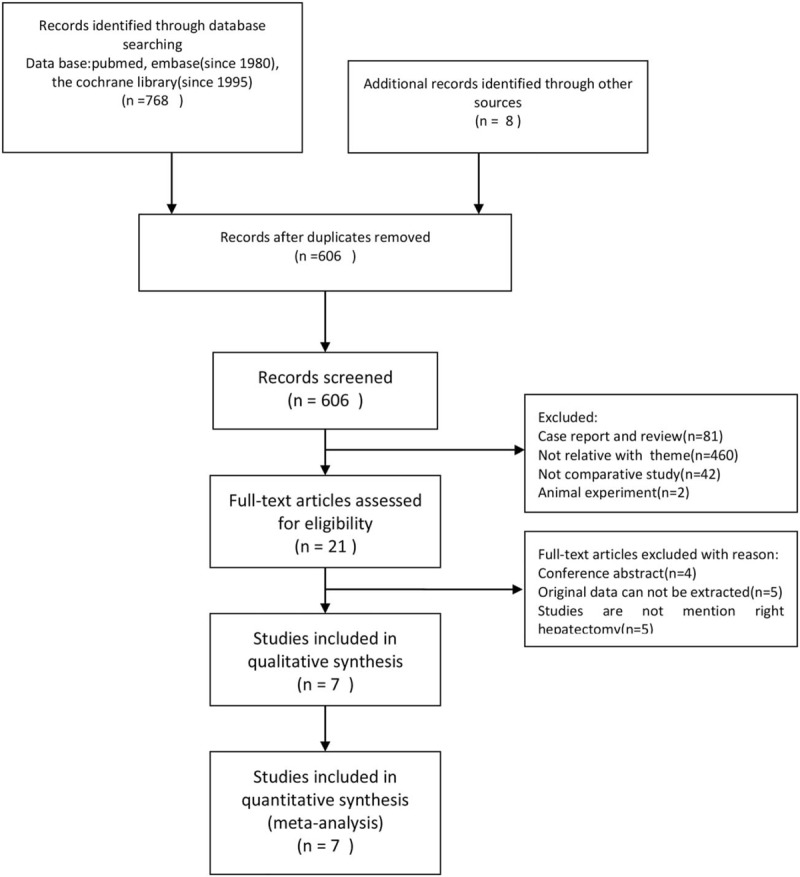
Preferred Reporting Items for Systematic Reviews and Meta-Analyses flow diagram.

Using the afore mentioned search strategy, 768 papers were identified from databases and an additional 8 papers were included from a manual search. After deleting 170 duplications, the article titles and abstracts of the remaining 606 papers were read carefully, of these, 597 studies were excluded. Omitted studies did not conform to our inclusion criteria Finally, 7 studies were included in our analysis.^[18–24]^ The strategy of literature inclusion is described in Figure 1, according to the Preferred Reporting Items for Systematic Reviews and Meta-Analyses (PRISMA) criteria.

### 3.1 Characteristics of the included studies

Characteristics of the 7 included studies, 4 of which were pair-matched studies, are listed in Table 1. The quality assessment of the included studies is summarized in Table 2. All 7 studies had a retrospective design with 467 patients in 2 groups (213 underwent LRH and 245 underwent ORH). The demographic features, preoperative comorbidity, and underlying liver diseases of the LRH group and ORH group were similar in each study. Only a laparoscopic technique was used in 5 studies.^[18,19,22–24]^ The other 2 studies^[20,21]^ enrolled patients who received the hand-assisted laparoscopic technique.

**Table 1:**
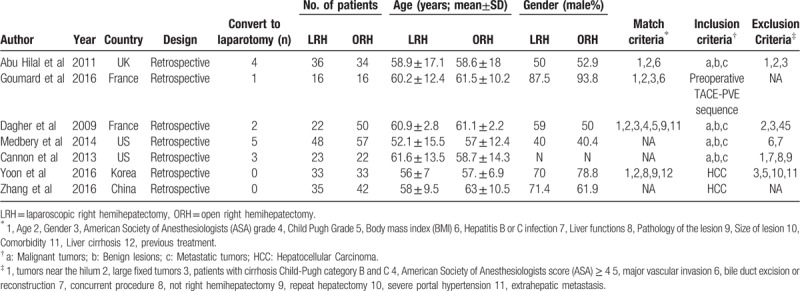
Patients’ characteristics of the included studies.

**Table 2:**
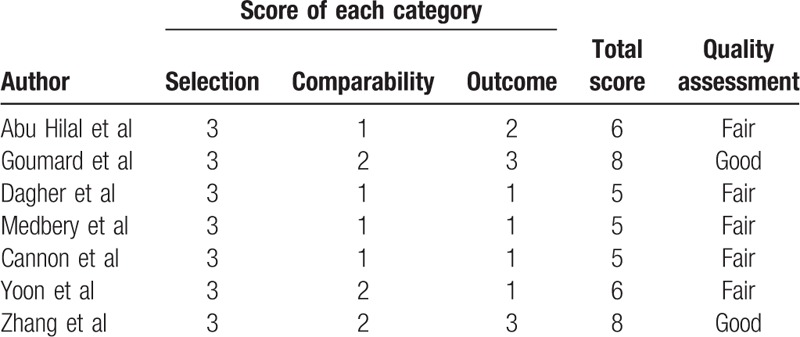
Quality assessment of the included studies via Newcastle–Ottawa Scale (NOS).

### 3.2 Results of the meta-analysis

Results of the overall meta-analysis, FDR and subgroup analysis for intraoperative and postoperative outcomes, including the operative time, blood loss, requirement for blood transfusion, hepatic portal occlusion, rate of R0 resection, overall postoperative complications, mild complications, severe complications, length of hospital stay, and hospital mortality, are listed in Table 3. *P* value is smaller than *P*_adjusted_ which demonstrate no false-positive result exit.

**Table 3:**
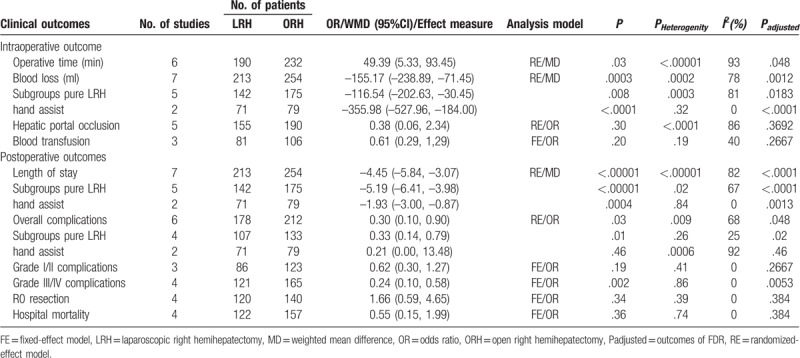
Pooled analysis and subgroup analysis of clinical outcome.

Here we use *P* value to describe the following results.

### 3.3 Intraoperative outcomes

#### 3.3.1 Mean operative time

**Figure 2:**
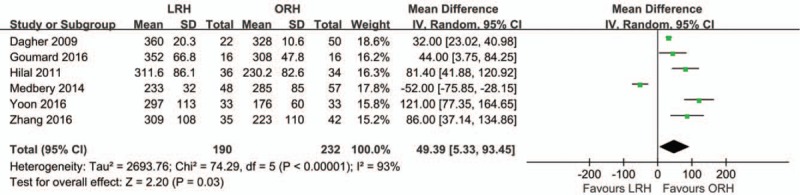
Forest plot showing mean operative time. A random effects model was used for meta-analysis. Mean Differences are shown with 95% CIs.

Six studies were included in the analysis of the mean operative time. High heterogeneity was observed (*I*^2^ = 93%). In the random-effect model, the mean operative time was significantly longer in the LRH group than in the ORH group (MD = 49.39; 95% CI, 5.33, 93.45; *Z* = 2.20; *P* = 0.03; Fig. 2).

#### 3.3.2 Intraoperative blood loss

**Figure 3:**
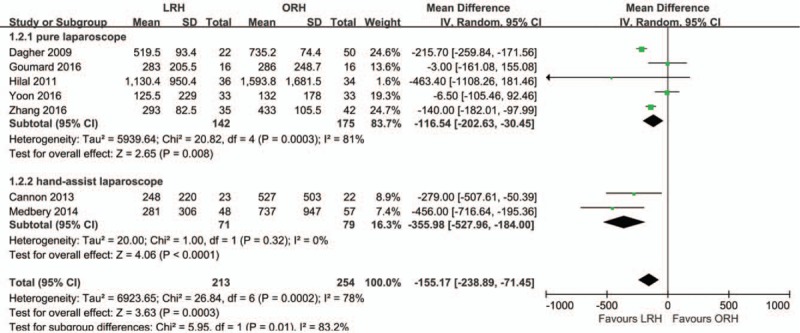
Forest plot showing intraoperative blood loss. A random effects model was used for meta-analysis. Mean Differences are shown with 95% CIs.

The analysis of intraoperative blood loss included all 7 studies. There was high heterogeneity among the studies (*I*^2^ = 78%). In the random-effect model, the intraoperative blood loss was significantly lower in the LRH group than in the ORH group (MD = –155.17; 95% CI, –238.89, –71.45; *Z* = 3.63; *P* = .0003; Fig. 3). The subgroup analysis indicated that intraoperative blood loss was significantly lower in the pure LRH group than in the ORH group (MD = –116.54; 95% CI, –202.63, –30.45; *Z* = 2.65; *P* = .008), with high heterogeneity (*I*^2^ = 81%). This result is consistent with that found in the hand-assisted laparoscopic subgroup analysis (MD = –355.98; 95% CI, –527.96, –184.00; *Z* = 4.06; *P* < .0001), without heterogeneity (*I*^2^ = 0%).

#### 3.3.3 Portal occlusion

The number of cases of portal occlusion was reported in 5 studies. There was high heterogeneity between the included studies (*I*^2^ = 86%). In the random-effect model, there was no significant difference in cases of portal occlusion between the LRH group and ORH group (OR = 0.38; 95% CI, 0.06, 2.34; *Z* = 1.04; *P* = .30).

#### 3.3.4 Intraoperative transfusion

The cases of blood transfusion were extracted from 3 studies. There was moderate heterogeneity among the studies (*I*^2^ = 40%). In the fixed-effect model, there was no significant difference in the percentage of transfusions between the LRH group and ORH group (OR = 0.61; 95% CI, 0.29, 1.29; *Z* = 1.29; *P* = .20).

### 3.4 Postoperative outcomes

#### 3.4.1 Length of stay

**Figure 4:**
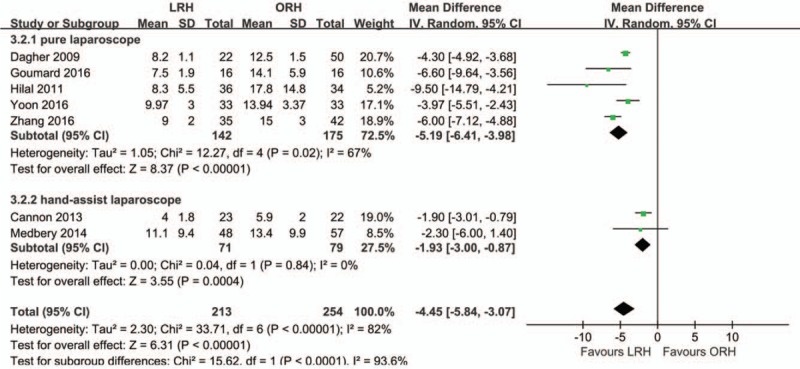
Forest plot showing length of stay. A random-effects model was used for meta-analysis. Mean Differences are shown with 95% CIs.

All 7 studies were included in the analysis of length of stay (LOS). There was high heterogeneity between included studies (*I*^2^ = 82%). In the random-effect model, the LOS was shorter in the LRH group than in the ORH group (MD = –4.45; 95% CI, –5.84, –3.07; *Z* = 6.31; *P* < .00001; Fig. 4). The result of subgroup analysis indicated that LOS was shorter by use of pure LRH and hand-assisted LRH manners than ORH, with reduced heterogeneity in the pure LRH group (*I*^2^ = 67%) and no heterogeneity in the hand-assisted LRH group (*I*^2^ = 0%).

#### 3.4.2 Overall complications

**Figure 5:**
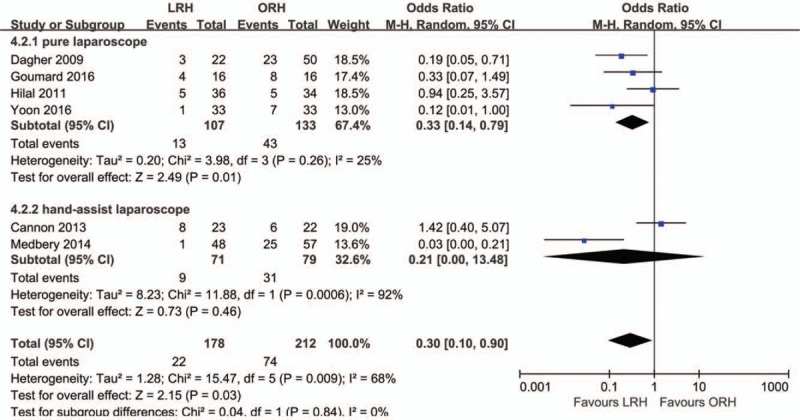
Forest plot showing overall complications. A random effects model was used for meta-analysis. Odds ratios are shown with 95% CIs.

Six studies were included in the analysis of overall complications. There was high heterogeneity of general complications among the studies (*I*^2^ = 68%). In the random-effect model, a significant difference was obtained (OR = 0.30; 95% CI, 0.10, 0.90; *Z* = 2.15; *P* = .03; Fig. 5). The incidence rate of overall complications was much lower in the LRH group than in the ORH group. The subgroup analysis indicated that the incidence rate of complications was lower in the pure LRH group than in the ORH group (OR = 0.33; 95% CI, 0.14, 0.79; *Z* = 2.49; *P* = .01), with low heterogeneity (*I*^2^ = 25%). In contrast, there was no significant difference between hand-assisted LRH group and ORH group.

#### 3.4.3 Mild and severe complications

**Figure 6:**
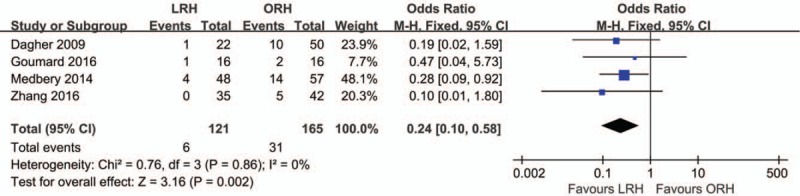
Forest plot showing severe complications. A Mantel–Haenszel fixed effects model was used for meta-analysis. Odds ratios are shown with 95% CIs.

Based on the Clavien-Dindo classification of postoperative complications,^[17,18]^ grades I-II and grades III-V were identified as mild and severe complications, respectively. Four studies were included in this analysis.^[18,20–22]^ For mild complications, no heterogeneity was observed among the studies (*I*^2^ = 0%). In the fixed-effect model, there was no visible difference between the LRH group and ORH group (OR = 0.62; 95% CI, 0.30, 1.27; *Z* = 1.31; *P* = .19). In contrast, with severe complications, there was no heterogeneity among the 3 included studies (*I*^2^ = 0%). However, in the fixed-effect model, there was a significant difference between these 2 groups (OR = 0.24; 95% CI, 0.10, 0.58; *Z* = 3.16; *P* = .002; Fig. 6). The numbers of cases of severe complications were significantly lower in the LRH group than in the ORH group.

#### 3.4.4 Postoperative mortality

Four studies were included in the analysis of postoperative mortality. There was no heterogeneity among the studies (*I*^2^ = 0%). In the fixed-effect model, there was no significant difference of postoperative morbidity in the LRH and ORH groups (OR = 0.55; 95% CI, 0.15,1.99; *Z* = 0.91; *P* = .36).

### 3.5 Rate of R0 resection

We compared the cases of R0 resection among 4 studies. There was no heterogeneity among the included studies (*I*^2^ = 0%). In the fixed-effect model, there was no significant difference in the percentage of transfusions between the LRH group and ORH group (OR = 1.66; 95% CI, 0.59, 4.65; *Z* = 0.96; *P* = .34).

### 3.6 Publication bias

Results of the Begg test (*P* = .764) and Egger test (*P* = .754) showed that no obvious publication bias existed in our analysis.

## 4 Discussion

Since Carl Langenbuch first successfully performed liver resection in 1888,^[25]^ this procedure has been prevalently applied for treating liver diseases. Nowadays, laparoscopic major hepatectomy, as an innovative technique of major liver resection, is a challenging operation limited by vision exposure, uncontrollable perioperative bleeding, and other technical difficulties. Only a few medical centers initially attempted to and reported promising outcomes of laparoscopic major hepatectomy.^[18,26,27]^ As the benefits of minimally invasive operation are increasingly reported, published cases of this procedure are rapidly increasing.^[28]^ Up to now, there is no definite evidence of safety and feasibility comparing these 2 surgical methods. Therefore, we conducted a meta-analysis to compare laparoscopic and ORH in operative details and postoperative course. To our knowledge, this is the first meta-analysis evaluating the benefits between LRH and ORH.

Our meta-analysis showed that the mean operative time in was significantly longer in the LRH group than in the ORH group. A lower intraoperative blood loss, lower morbidity rate, and shorter LOS were observed in the laparoscopic group than in the ORH group. In addition, the transfusion rate, R0 resection rate, rate of portal occlusion, mild postoperative complications, and postoperative mortality were similar between the LRH group and ORH group.

The selection criteria of patients are crucial for successfully performing right hepatectomy. Liver function is a determinant regardless of the technique and surgical skill. Patients with decompensated liver function are often associated with a vulnerable body condition, which may lead to intraoperative bleeding and anesthetic accident.^[18,29]^ Thus, a Child A score must be achieved preoperatively. No major vascular invasion is essential for radical operation. It is challenging to perform laparoscopic operation in patients with a large subcapsular tumor. The limited space and mobilization of the liver may cause the tumor break up and spread. For the same reason, patients with a tumor close to main vessels are considered unsuitable for laparoscopic operation.^[30]^ Besides, patients with a history of abdominal operation are contraindicated for laparoscopic operation.^[31,32]^ The above selection criteria caused the selection bias of the included patients, so the results of this study were biased to a certain extent.

To maximally decrease ischemia-reperfusion injury, portal occlusion is currently performed instead of total hepatic vascular occlusion. No statistical difference was found in the rate of using portal occlusion between the 2 study groups. Medbery et al^[21]^ reported that LRH can be achieved successfully without the need for portal occlusion. By using the laparoscopic technique, the structure and vessel organization can be clearly visualized, so the operation becomes easier and more accurate, thereby decreasing the risk of bleeding. At our clinical center, portal occlusion is replaced by regional hepatic vascular occlusion, wherein the porta hepatis structures are precisely divided.^[29]^ After regional control of liver inflow and outflow, the ischemic line will appear.

Our results showed that the loss of blood was significantly lower in the LRH group than in the ORH group. The subgroup analysis showed a consistent result: blood loss was also lower in the pure LRH and hand-assisted LRH groups than in the ORH group. According to previous experience, the benefits of portal occlusion by using laparoscopy have been obtained.^[29]^ Using the laparoscope, surgeons can see tissue more clearly and approach structures more accurately. Besides, the pressure generated by pneumoperitoneum helps decrease bleeding during liver parenchyma resection. Regarding the transfusion rate, no difference was detected between the 2 study groups, which was contradictory with the result that intraoperative bleeding was significantly lower in the LRH group than in the ORH group. In our viewpoint, blood loss is not the only reason for transfusion; the perioperative body condition must be considered, for example, the lower preoperative hemoglobin level, older age, prior systemic chemotherapy, and lower preoperative nutritional parameters.^[33]^ In addition, patient selection bias is also responsible for this result.

Concerning the operative time, it differed from each study. Six studies^[18–22,24]^ were included in this analysis, 5^[18–20,22,24]^ of which reported a longer operative time in the LRH group than in the ORH group. One study reported by Medbery et al^[21]^ showed a shorter operative time in the LRH group than in the ORH group. The mean operative times were 233 minutes using the hand-assisted technique and 285 minutes with laparotomy. Kluger et al^[34]^ from New York Presbyterian Hospital presented a learning curve that was similar to our experience^[29]^; they showed a decreasing time of operation that was finally getting close to that for laparotomy. With emerging equipment such as a robotic-assistant system and improvement of the laparoscopic technique, we speculate that the operative time will be much shorter with minimally invasive operation.

There were significantly fewer overall postoperative complications in the LRH group than in the ORH group, which was consistent with that reported in a previous study.^[35]^ Additionally, the results of subgroup analysis showed that the incidence rate of overall complications was lower in the pure laparoscopy group, whereas no difference was detected in the hand-assisted laparoscopy group; this can be explained by the fact that the hand ports used in the hand-assisted technique cause more surgical wounds to patients compared with pure laparoscopic operation. It also means that pure laparoscopy can greatly reduce operative trauma and surgical stress, which lead to fewer postoperative complications and it responds to the call to the modern concept of fast track operation.^[36]^ It is worth mentioning that a significant difference in grade III/IV postoperative complications was detected between the LRH group and ORH group. There were fewer severe complications in the LRH group than in the ORH group, but there was no difference between groups in mild complications. Hence, we can speculate that LRH does not contribute to reducing mild complications, but it does reduce the incidence rate of severe complications.

In addition, the LOS was much shorter in the LRH group than in the ORH group. There was no statistical difference in the incidence rate of postoperative mortality between the LRH group and ORH group. Regarding postoperative mortality, 4 studies^[18,21,22,24]^ reported that 8 patients died (liver failure, 2; sepsis, 2; liver failure and sepsis,1; acute respiratory distress syndrome and upper gastrointestinal bleeding, 1; respiratory failure, 1; and stroke, 1). These results demonstrated that laparoscopic operation does not increase the risk of postoperative mortality in right hepatectomy; nonetheless, surgeons should focus more on the perioperative liver function and infection control.

Overall, LRH has some promising short-term outcomes in terms of intraoperative bleeding, postoperative severe complications, and LOS. Additionally, right hepatectomy can achieve an equally pathological rate of R0 resection using a minimally invasive technique compared with the conventional open technique. With the learning curve, surgeons can achieve similar outcomes by using the laparoscopic technique in contrast with the conventional open technique. LRH can be considered a feasible choice for treating liver tumors in select patients, although more evidence is needed to make a definitive conclusion.

The sensitivity analysis showed that after excluding studies on the pooled outcomes ordinally, the statistical results and the heterogeneity have no obvious difference.

Certainly, there are still some limitation underlying this meta-analysis. First, given that no RCTs were included in this meta-analysis, we must presume that the underlying risks of bias remain high. The quality assessment of 5 included articles is fair, which may affect the effectiveness of the meta-analysis. Secondly, high heterogeneity existed in the pooled analysis of some outcomes, even although we reviewed the studies carefully and found no obvious clinical heterogeneity or diverse indications of operation between the studies. We speculated that the high heterogeneity could be attributed to the subjectivity in the process of measuring the data. Therefore, our results must be interpreted carefully, although the random-effect model was used for this meta-analysis. Thirdly, some aspects of the clinical outcomes, such as the length of intensive care unit stay and postoperative paregoric use, could not be analyzed in this meta-analysis because of the lack of sufficient data. Fourthly, the included studies which were reported by specialized and high-volume centers contributed to the promising results. Inexperienced surgeons may not achieve such an expected results. What is more, the postoperative course is very program dependent and variables can be exist. Finally, the Second International Consensus Conference held in Morioka^[2]^ recommends researchers obtain more evidence since laparoscopic major liver resection is still an innovative procedure in the exploration phase.

## 5 Conclusions

Various short-term advantages were identified in this meta-analysis, indicating that LRH can be considered a feasible choice in routine clinical practice. Nevertheless, we cannot draw definite conclusions from the results since no high-quality evidence was included in this meta-analysis. More well designed RCTs with an adequate sample size and extensive follow-up are necessary to make a definitive conclusion in the future.

## Author contributions

**Conceptualization:** Jianjun Wang.

**Data curation:** Yong Wang, Zhangqiang Wu, Junchao Zhou.

**Formal analysis:** Yong Wang, Tao Liu.

**Methodology:** Qiang Hong.

**Software:** Baojuan Fu, Qin Tong, Tao Liu, Zhangqiang Wu.

**Supervision:** Baojuan Fu.

**Writing – original draft:** Yuejun Fang, Qin Tong, Junchao Zhou.

**Writing – review & editing:** Qiang Hong.

## References

[1] Goumard C Farges O Laurent A An update on laparoscopic liver resection: the French Hepato-Bilio-Pancreatic Surgery Association statement. J Visc Surg 2015;152:107–12. 25753081 10.1016/j.jviscsurg.2015.02.003

[2] Wakabayashi G Cherqui D Geller DA Recommendations for laparoscopic liver resection: a report from the second international consensus conference held in Morioka. Ann Surg 2015;261:619–29. 25742461 10.1097/SLA.0000000000001184

[3] Long TC Bac NH Thuan ND Laparoscopic liver resection: 5-year experience at a single center. Surg Endosc 2014;28:796–802. 24196550 10.1007/s00464-013-3259-y PMC3931927

[4] Tranchart H Dagher I Laparoscopic liver resection: a review. J Visc Surg 2014;151:107–15. 24365035 10.1016/j.jviscsurg.2013.10.003

[5] Vigano L Tayar C Laurent A Laparoscopic liver resection: a systematic review. J Hepato-biliary-pancreatic Surg 2009;16:410–21. 10.1007/s00534-009-0120-8 19495556

[6] Abu Hilal M Pearce NW Laparoscopic left lateral liver sectionectomy: a safe, efficient, reproducible technique. Dig Surg 2008;25:305–8. 18784413 10.1159/000155222

[7] Buell JF Thomas MT Rudich S Experience with more than 500 minimally invasive hepatic procedures. Ann Surg 2008;248:475–86. 18791368 10.1097/SLA.0b013e318185e647

[8] Koffron A Geller D Gamblin TC Laparoscopic liver surgery: shifting the management of liver tumors. Hepatology (Baltimore, Md) 2006;44:1694–700. 10.1002/hep.21485 17133494

[9] Nguyen KT Geller DA Is laparoscopic liver resection safe and comparable to open liver resection for hepatocellular carcinoma? Ann Surg Oncol 2009;16:1765–7. 19412631 10.1245/s10434-009-0496-3 PMC2695865

[10] Chang S Laurent A Tayar C Laparoscopy as a routine approach for left lateral sectionectomy. Br J Surg 2007;94:58–63. 17054316 10.1002/bjs.5562

[11] Hackl C Schlitt HJ Renner P Liver surgery in cirrhosis and portal hypertension. World J Gastroenterol 2016;22:2725–35. 26973411 10.3748/wjg.v22.i9.2725 PMC4777995

[12] Machado MA Makdissi FF Surjan RC Totally laparoscopic right hepatectomy with roux-en-y hepaticojejunostomy for hilar cholangiocarcinoma. HPB 2014;16:707. 24522989 10.1245/s10434-014-3517-9

[13] Choi SH Choi GH Han DH Laparoscopic right hepatectomy: toward protocolization and simplification. Ann Surg Oncol 2017;24:554–5. 27682999 10.1245/s10434-016-5562-z

[14] Gayet B Cavaliere D Vibert E Totally laparoscopic right hepatectomy. Am J Surg 2007;194:685–9. 17936436 10.1016/j.amjsurg.2006.11.044

[15] Wells GA Shea B O’Connell D The Newcastle-Ottawa Scale (NOS) for assessing the quality if nonrandomized studies in meta-analyses. 2011. Available at: http://www.ohri.ca/programs/clinical_epidemiology/oxford.asp [access date November 25, 2016].

[16] Hozo SP Djulbegovic B Hozo I Estimating the mean and variance from the median, range, and the size of a sample. BMC Med Res Methodol 2005;5:13. 15840177 10.1186/1471-2288-5-13 PMC1097734

[17] Engels EA Schmid CH Terrin N Heterogeneity and statistical significance in meta-analysis: an empirical study of 125 meta-analyses. Stat Med 2000;19:1707–28. 10861773 10.1002/1097-0258(20000715)19:13<1707::aid-sim491>3.0.co;2-p

[18] Dagher I Di Giuro G Dubrez J Laparoscopic versus open right hepatectomy: a comparative study. Am J Surg 2009;198:173–7. 19268902 10.1016/j.amjsurg.2008.09.015

[19] Yoon YI Kim KH Kang SH Pure laparoscopic versus open right hepatectomy for hepatocellular carcinoma in patients with cirrhosis: a propensity score matched analysis. Ann Surg 2017;265:856–63. 27849661 10.1097/SLA.0000000000002072

[20] Zhang Y Chen XM Sun DL Short-term outcomes of laparoscopic versus open right hemihepatectomy for hepatocellular carcinoma. Surg Laparosc Endosc Percutan Tech 2016;26:e157–60. 27846174 10.1097/SLE.0000000000000355

[21] Medbery RL Chadid TS Sweeney JF Laparoscopic vs open right hepatectomy: a value-based analysis. J Am Coll Surg 2014;218:929–39. 24680574 10.1016/j.jamcollsurg.2014.01.045

[22] Goumard C Komatsu S Brustia R Technical feasibility and safety of laparoscopic right hepatectomy for hepatocellular carcinoma following sequential TACE-PVE: a comparative study. Surg Endosc 2017;31:2340–9. 27655376 10.1007/s00464-016-5225-y

[23] Cannon RM Scoggins CR Callender GG Financial comparison of laparoscopic versus open hepatic resection using deviation-based cost modeling. Ann Surg Oncol 2013;20:2887–92. 23636514 10.1245/s10434-013-2993-7

[24] Abu Hilal M Di Fabio F Teng MJ Single-centre comparative study of laparoscopic versus open right hepatectomy. J Gastrointest Surg 2011;15:818–23. 21380633 10.1007/s11605-011-1468-z

[25] Rains AJ A thought for Carl Langenbuch (1846-1901): a centenary. Ann R Coll Surg Engl 1982;64:268–9. 7046607 PMC2494149

[26] Pearce NW Di Fabio F Teng MJ Laparoscopic right hepatectomy: a challenging, but feasible, safe and efficient procedure. Am J Surg 2011;202:e52–8. 21861979 10.1016/j.amjsurg.2010.08.032

[27] Borzellino G Ruzzenente A Minicozzi AM Laparoscopic hepatic resection. Surg Endosc Other Interv Tech 2006;20:787–90. 10.1007/s00464-004-2186-3 16544083

[28] Buell JF Cherqui D Geller DA The international position on laparoscopic liver surgery: the Louisville Statement, 2008. Ann Surg 2009;250:825–30. 19916210 10.1097/sla.0b013e3181b3b2d8

[29] Cai X Li Z Zhang Y Laparoscopic liver resection and the learning curve: a 14-year, single-center experience. Surg Endosc 2014;28:1334–41. 24399518 10.1007/s00464-013-3333-5

[30] Otto G Heise M Hoppe-Lotichius M Hilar cholangiocarcinoma: right versus left hepatectomy. Zentralbl Chir 2012;137:535–40. 23264194 10.1055/s-0032-1328024

[31] Amikura K Sakamoto H Takahashi A Useful device for hepatectomy in patients with a surgical history of bile duct-GI tract anastomosis at the porta hepatis. Gan To Kagaku Ryoho 2014;41:1497–9. 25731231

[32] Matsukawa H Shiozaki S Satoh D Experiences of laparoscopic repeat hepatectomy for recurrent hepatic cancer after open hepatectomy. Gan To Kagaku Ryoho 2014;41:2098–100. 25731435

[33] Wehry J Cannon R Scoggins CR Restrictive blood transfusion protocol in liver resection patients reduces blood transfusions with no increase in patient morbidity. Am J Surg 2015;209:280–8. 25305797 10.1016/j.amjsurg.2014.06.016 PMC5812266

[34] Kluger MD Vigano L Barroso R The learning curve in laparoscopic major liver resection. J Hepatobiliary Pancreat Sci 2013;20:131–6. 23064988 10.1007/s00534-012-0571-1

[35] Yin Z Fan X Ye H Short- and long-term outcomes after laparoscopic and open hepatectomy for hepatocellular carcinoma: a global systematic review and meta-analysis. Ann Surg Oncol 2013;20:1203–15. 23099728 10.1245/s10434-012-2705-8

[36] Liang X Ying H Wang H Enhanced recovery program versus traditional care in laparoscopic hepatectomy. Medicine (Baltimore) 2016;95:e2835. 26937913 10.1097/MD.0000000000002835 PMC4779010

